# Irinotecan plus gemcitabine *vs* irinotecan for the second-line treatment of patients with advanced non-small-cell lung cancer pretreated with docetaxel and cisplatin: a multicentre, randomised, phase II study

**DOI:** 10.1038/sj.bjc.6602010

**Published:** 2004-07-06

**Authors:** V Georgoulias, C Kouroussis, A Agelidou, I Boukovinas, P Palamidas, E Stavrinidis, A Polyzos, K Syrigos, M Veslemes, M Toubis, A Ardavanis, E Tselepatiotis, I Vlachonikolis

**Affiliations:** 1 Department of Medical Oncology, University General Hospital of Heraklion, Greece; 2 First Department of Pulmonary Diseases, ‘Sismanoglion’ General Hospital, Greece; 3 Second Department of Medical Oncology, ‘Theagenion’ Anticancer Hospital, Thessaloniki, Greece; 4 Second Department of Pulmonary Diseases, Sismanoglion' General Hospital of Athens, Greece; 5 First Department of Medical Oncology, ‘Agios Savvas’ Anticancer Hospital, Athens, Greece; 6 Oncology Unit, Department of Propedeutic Medicine, University of Athens, Greece; 7 Medical Oncology Unit, Third Department of Internal Medicine, University of Athens, Greece; 8 Department of Pulmonary Diseases, University of Athens, Greece; 9 Eight Department of Pulmonary Diseases, ‘Sotiria’ Hospital of Athens, Greece; 10 Department of Internal Medicine, ‘Patision’ General Hospital of Athens, Greece; 11 Department of Biostatistics, School of Medicine, University of Crete, Greece

**Keywords:** NSCLC, second-line chemotherapy, CPT-11, gemcitabine

## Abstract

To compare irinotecan (CPT-11)+gemcitabine *vs* CPT-11 alone as second-line treatment for patients with advanced non-small cell lung cancer (NSCLC) progressing after docetaxel–cisplatinum-based therapy. A total of 147 evaluable, pretreated patients, with NSCLC, received either gemcitabine (1000 mg m^−2^, days 1 and 8)+CPT-11 (300 mg m^−2^, day 8) (Group A, *n*=76) or CPT-11 (300 mg m^−2^, day 1) (Group B, *n*=71), every 3 weeks. All patients were evaluable for response and toxicity. The objective response rate was 18.4% (95% CI: 9.71–27.14%) and 4.2% (95% CI: 0–8.90%) (*P*=0.009) for groups A and B, respectively. No significant differences between the two groups in terms of the median duration of response, time to tumour progression, overall survival and 1-year survival were observed. The CPT-11/gemcitabine regimen significantly improved the patients' quality of life (‘general mood today’ (*P*=0.014), ‘coughing’ (*P*=0.003) and ‘intensity of symptoms’ (*P*=0.034)) compared with CPT-11. More cycles had to be delayed (*P*=0.001) and required prophylactic growth factor support (*P*=0.001) in Group A than B. Three (3.9%) patients in Group A and eight (11.3%) in Group B developed febrile neutropenia (*P*=0.09); one patient died of sepsis in each group. Three additional (Group A, *n*=1; Group B, *n*=2) treatment-related deaths were observed. Grade 3–4 haematologic toxicity was comparable in the two groups except anaemia (*P*=0.03 in favour of CPT-11). Other nonhaematologic toxicities were mild and similar in the two groups. CPT-11+gemcitabine resulted in a higher response rate and better control of disease-related symptoms than CPT-11 alone, but without any improvement in the overall survival.

Cisplatin-based chemotherapy confers a survival advantage and still represents the standard of care in inoperable locally advanced and metastatic non-small-cell lung cancer (NSCLC) (Souquet *et al*, 1993; NSCLC Collaborative Group, 1995) and its use is increased as front-line treatment (Belani, 1993) therefore, a higher proportion of patients are becoming candidates for second-line chemotherapy.

Several phase II studies have shown that some newer drugs either alone or in combination might be active in the second-line setting (Fossella *et al*, 1997; Androulakis *et al*, 1998; Ferrigno and Buccheri, 2000; Huisman *et al*, 2000; Iaffaioli *et al*, 2000). Randomised studies demonstrated that second-line docetaxel conferred survival and clinical benefit as well as improved quality of life compared with best supportive care (Shepherd *et al*, 2000) or monotherapy with vinorelbine or ifosfamide (Fossella *et al*, 2000). Also, the pyrimidine antimetabolite gemcitabine has demonstrated significant activity in pretreated patients with advanced NSCLC, with objective responses ranging from 7 to 20%, and a median survival of 22–36 weeks (Baas *et al*, 1999; Crino *et al*, 1999; Reddy *et al*, 1999; Rossi *et al*, 1999). The data for irinotecan (CPT-11), a semisynthetic derivative of the plant alkaloid camptothecin (Kunimoto *et al*, 1987), are conflicting. In one study, CPT-11 failed to demonstrate any antitumour activity in 26 pretreated patients with NSCLC, whereas in another an overall response rate of 14% was achieved (Ferrigno and Buccheri, 2000).

Gemcitabine and CPT-11 have shown synergistic *in vitro* activity (Pei *et al*, 1997; Schwartz *et al* 2001). Their combination is both feasible and well tolerated (Lima *et al*, 1998; O'Reilly *et al*, 1999; Kakolyris *et al*, 2002). In a phase I study, it was revealed that the main dose-limiting events of the gemcitabine and CPT-11 combination given in a 3-weekly schedule were grade 4 thrombocytopenia, grade 3 diarrhoea and grade 3 asthenia (Kakolyris *et al*, 2002). In a pilot phase II study, where gemcitabine was used at a dose of 1000 mg m^−2^ in order to reduce the incidence of thrombocytopenia, we observed five PRs among 23 pretreated patients with docetaxel and cisplatin. The main toxicities included grade 3–4 diarrhoea in 20% and grade 3–4 neutropenia in 53% of the cycles.

As a consequence of the promising activity demonstrated by CPT-11 in combination with gemcitabine, we conducted a prospective, multicentre, randomised phase II study to compare the efficacy and tolerance of this regimen with that of CPT-11 monotherapy. CPT-11 monotherapy was chosen for the second arm for the following reasons: (i) all patients were pretreated with docetaxel and platinum; and (ii) to more appropriately evaluate the antitumour activity of CPT-11 in the second-line setting.

## PATIENTS AND METHODS

### Patients

Patients (aged ⩾18 years) with WHO performance status (PS) 0–2 and stage IIIB or IV, cytologically or histologically confirmed NSCLC, were enrolled into this trial. Patients progressing either under docetaxel/platinum (cisplatin or carboplatin) chemotherapy or after its completion were eligible for randomisation. The additional inclusion criteria were: at least one bidimensionally measurable lesion outside an irradiation field; adequate bone, kidney and liver function (with the exception of alkaline phosphatase, which could be up to five times the UNL in case of liver metastases). Prior radiotherapy was allowed, provided that it had been completed at least 4 weeks prior to enrolment and ⩽25% of the bone marrow had been irradiated; at least 4 weeks had to have elapsed from completion of the last cycle of front-line chemotherapy. Patients were excluded if there was severe cardiopulmonary insufficiency, a positive pregnancy test for women of child-bearing age, severe angina pectoris or a recent myocardial infarction, active infection or severe malnutrition (loss of >15% of body weight). All patients had to provide written, informed consent. The trial was approved by the Ethics and Scientific Committees of the participating institutions.

### Treatment plan and dose modifications

Eligible patients were stratified according to their PS (WHO 0–1 *vs* 2) and their best response to docetaxel/platinum first-line chemotherapy (CR+PR *vs* SD+PD); patients were randomised centrally to receive either gemcitabine (Gemzar®, Elli Lilly, Indianapolis, USA), 1000 mg m^−2^, as a 30-min intravenous (i.v.) infusion on days 1 and 8 and CPT-11 (Campto®, Aventis Pharma, Bridgewater, USA) 300 mg m^−2^, as a 60-min i.v. infusion on day 8, following the gemcitabine administration (Group A) or CPT-11 at the same dose on day 1 (Group B). Cycles were repeated every 3 weeks. All patients received prophylactic antiemetic therapy (odansetron 16 mg+dexamethasone 8 mg, given i.v.). Delayed diarrhoea due to CPT-11 was treated with loperamide.

Dose modifications were performed according to the haematological and gastrointestinal toxicity. Patients developing grade 3–4 neutropenia without fever received the subsequent cycles with prophylactic recombinant human-granulocyte colony-stimulating factor (rhG-CSF: Granocyte, Aventis Pharma) (150 *μ*g m^−2^, d_9_–d_15_). If grade 3–4 neutropenia recurred despite prophylactic rhG-CSF or febrile neutropenia (fever >37.5°C for at least 24 h with neutropenia grade 3–4) developed, the d_8_ doses of both drugs were reduced by 25% for all subsequent cycles. Patients with grade 4 thrombocytopenia were treated in subsequent cycles with a 25% reduction of the gemcitabine dose. Patients who presented with grade 3–4 delayed diarrhoea were treated in all subsequent cycles with a 25% reduction of the CPT-11 dose. Patients requiring more than one dose reduction were withdrawn from the study.

### Baseline and follow-up assessments

Baseline assessments included complete medical history and physical examination, complete blood cell count with differential and serum chemistry. Bidimensionally measurable lesions were determined by standard imaging procedures at baseline (chest X-ray, ultrasound, CT scans of the thorax, abdomen and brain, magnetic resonance imaging and whole body bone scan). Tumour assessments for response were performed every three chemotherapy cycles. Complete medical history and physical examinations, as well as complete blood cell count with differential and serum chemistry, were performed every 3 weeks. Chest X-rays were performed every two chemotherapy cycles. Treatment-related haematological toxicity was evaluated twice a week and daily in patients with grade 3**–**4 neutropenia or thrombocytopenia.

Patients who received at least three cycles of chemotherapy were assessed for response according to the WHO criteria (WHO, 1979). All responses, which had to be maintained for at least 4 weeks, were confirmed by an independent panel of radiologists. Patients who received at least one chemotherapy cycle were assessable for toxicity (WHO, 1979).

For the quality of life assessment, the Lung Cancer Symptom Scale (LCSS) and the EuroQOL (EQ-5D) questionnaire were used at baseline and every three chemotherapy cycles thereafter (Hollen *et al*, 1993; Rabin and de Charro, 2001).

### Statistical considerations

This was a prospective, multicentre, randomised phase II trial. The primary end point was the comparison of the median survivals for the two groups. Secondary end points included objective tumour response rates, duration of response, time to progression (TTP), treatment tolerance and quality of life. For the sample size calculation, a median survival of 9 and 5 months for Groups A and B, respectively, was assumed. A total of 72 patients arm^−1^ were required in order that the study has significant difference (at the 5% level) between the two survival curves with a power of 90%.

Differences of rates between groups were assessed by Pearson's *χ*^2^ test or Fisher's test where appropriate. Time-to-event end points were calculated using Kaplan–Meier methods with appropriate censoring (Collet, 1999). The independent influence of several factors on the risk of nonresponse, relapse or nonsurvival was assessed by logistic regression, while that on the hazards of relapse or failure of survival by Cox's proportional-hazards model. Survival was calculated from the date of randomisation to second-line chemotherapy until the date of death. Time to disease progression was assessed from the date of randomisation until the date of disease progression. Response duration was calculated from the date that the criteria of response were met for the first time until the date of documentation of disease progression.

## RESULTS

### Patient demographics

From August 1999 to April 2002, 154 pretreated patients with NSCLC were registered and randomised to receive either CPT-11/gemcitabine (Group A, *n*=79) or CPT-11 (Group B, *n*=75). Three patients in Group A (two never received chemotherapy and one because of a major protocol violation) and four patients in Group B (two never received chemotherapy, one was treated with RT because of superior vena cava syndrome and one for a major protocol violation) were not evaluable. The baseline patient characteristics are presented in Table 1

**Table 1:** Patient characteristics

	**CPT-11/GEM (Group A)**		**CPT-11 (Group B)**	
	**No. of patients**	**%**	**No. of patients**	**%**
Patients randomised	79		75	
Evaluable for toxicity	76		71	
Evaluable for response	76		71	
				
*Gender*				
Male	71	93	63	89
Female	5	7	8	11
				
*Age (years)*				
Median (range)	60.5 (42–72)		64 (42–72)	
				
*Performance status (WHO)*				
0–1	69	91	64	90
2	7	9	7	10
				
*Histology*				
Adenocarcinomas	25	33	24	34
Nonadenocarcinomas	51	67	47	66
				
*Stage*				
i.v.	76	100	71	100
				
*No. of organs involved*				
1	28	37	28	40
2	32	42	33	47
⩾3	16	21	10	13
				
*Prior treatment*				
Radiotherapy	12	15	17	23
Chemotherapy	100	100	100	100
				
*Platinum compound*				
CDDP	59	78	58	82
Carboplatin	17	22	13	18
				
*Best response to first-line chemoTx*				
CR/PR	27	36	19	27
SD/PD	49	64	52	73

WHO=World Health Organisation; i.v.=intravenous; CPT-11=irinotecan; GEM=gemcitabine; CDDP=cisplatin; CR=complete response; PR=partial response; SD=stable disease; PD=progressive disease.

. The two groups were well balanced with respect to gender, PS, histology and extension of the disease.

### Response to treatment

In total, 76 patients in Group A and 71 patients in Group B were assessed for response. Three Group A and two Group B patients were not evaluable, but were considered as progressors in the intention-to-treat analysis. In all, 14 PRs (ORR=18.4%; 95% CI: 9.7–27.1%) were observed in Group A and one (1.4%) CR and two (2.8%) PRs (ORR=4.2%; 95% CI: 0–8.9%) in Group B (*P*=0.009; Table 2

**Table 2:** Efficacy of CPT-11+GEM regimen and CPT-11 alone as second-line treatment of NSCLC patients pretreated with docetaxel and CDDP

	**CPT-11+GEM (Group A)**	**CPT-11 (Group B)**	***P*-value**
CR	—	1 (1.4%)	
PR	14 (18.4%)	2 (2.8%)	
ORR (CR+PR)	14 (18.4%)	3 (4.2%)	0.009^a^
(95% CI)	9.71–27.14%	−0.45–8.90%	
SD	20 (26.3%)	18 (25.3%)	
PD	42 (55.3%)	50 (70.4%)	
			
*Duration of response (mo)*			
Median (range)	5.5 (2–14)	4.0 (2–12)	0.885
			
*Time to progression (mo)*			
Median (range)	7.5 (2.5–19)	5.0 (1.0–15.5)	0.346
			
*Survival (mo)*			
Median (range)	9.0 (0.5–29)	7.0 (0.5–24)	0.589
			
1-year survival (%)	24.5	29	

NSCLC=non-small-cell lung cancer; mo=months; CI=confidence interval.

a Fisher's exact test.

). A total of 20 (26.3%) Group A and 18 (25.3%) Group B patients had SD, while 42 (55.3%) and 50 (70.4%) Group A and B patients, respectively, had PD. The risk of nonresponse (SD+PD) among Group B patients was about four times (odds ration: 3.902; 95% CI: 1.032–14.75) that of Group A; conversely, age, gender, PS, histology or number of involved sites had no significant effect on the risk of nonresponse. Of the 14 responders in Group A, three had experienced a PR, six SD and five PD on first-line chemotherapy. Of the three responders in Group B, one had a PR, one SD and one PD on front-line chemotherapy. The objective response rate for CPT-11/gemcitabine was significantly higher than that for CPT-11 alone in the lung (18 *vs* 5%; *P*=0.017), liver (33 *vs* 0%; *P*=0.031) and lymph nodes (27 *vs* 6%; *P*=0.031). The median duration of response was 5.5 and 4 months in Groups A and B, respectively (Table 2).

The median interval from the last first-line chemotherapy treatment for responders *vs* nonresponders in Group A was 1.25 (range 1–29) and 3 (range 1–24) months, respectively (*P*=0.314); in Group B patients, the corresponding values were 1 (range, 1–6) and 3 (range, 1–42) months, respectively (*P*=0.315).

Out of 34 Group A and 17 (81%) out of 21 Group B patients, 27 (79.4%)who had CR, PR and SD during treatment had disease recurrence during the follow-up period (*P*=0.890). The median TTP was 7.5 (range, 2.5–19) and 5.0 (range, 1.0–15.5) months for Group A and B patients, respectively (*P*=0.346; Table 2). A total of 21 (28%) and 15 (21.1%) patients in Groups A and B, respectively, received third-line chemotherapy. The median overall survival of patients who received third-line chemotherapy was 10 (range, 2.5–29) months compared with 6 (range, 0.5–24) months survival of patients who did not received further treatment (log-rank test: *P*=0.0065).

### Survival

The median follow-up period was 7.5 (range, 0.5–29) months for Group A and 6.5 (range, 0.5–24) months for Group B patients. A total of 54 (71%) Group A and 53 (75%) Group B patients died (*P*=0.625). The causes of death were disease progression (Group A, *n*=50; Group B, *n*=49), treatment-related toxicity (Group A, *n*=2; Group B, *n*=3) and nontreatment-related reasons (Group A: one patient with pulmonary embolism and one patient with postorthopedic haemorrhage; Group B: one with myocardial infarction and one with bowel obstruction).

**Figure 1:**
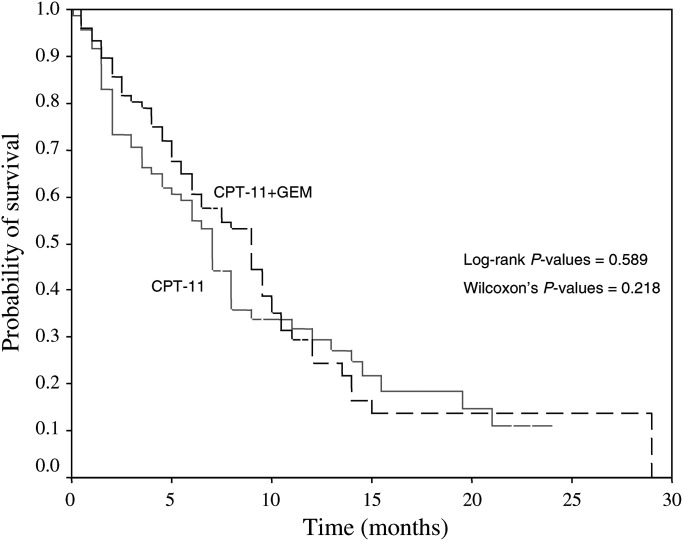
Kaplan–Meier estimate of the survival for Group A (CPT-11+gemcitabine; dotted line) and Group B (CPT-11; continuous line) patients.

The median survival time was 9 (range, 0.5–29) months in Group A and 7 (range, 0.5–24) months in Group B patients (*P*=0.589; Figure 1

, Table 2). The 1-year survival rates were 24.5 and 29% for Group A and B patients. Taken all patients together, survival was significantly affected by the PS (*P*=0.047) but not by the age, gender, tumour histology or number of involved sites. Overall, the median survival among patients with PS 0–1 was 8 (range, 0.5–29) months compared to 1.5 (range 0.5–23) months for patients with PS 2. PS had an independent effect on survival since the hazard of death of patients with PS 2 was about two times that of patients with PS 0–1 (HR: 1.853; 95% CI: 1.183–2.990). The median survival for Group A patients with PS 0–1 and 2 were 9 (range, 0.5–29) and 1.5 (range, 0.5–16.5) months, respectively (Wilcoxon's test; *P*=0.033); the corresponding values for Group B patients were 7 (range, 0.5–24) and 1.5 (range, 0.5–23) months (Wilcoxon's test; *P*<0.001).

### Compliance with the treatment

A total of 291 and 243 chemotherapy cycles were administered to Group A (median three cycles; range 1–9) and Group B (median three cycles; range, 1–8) patients, respectively. The median interval between cycles in Groups A and B was 23 (range, 21–37) and 21 (range, 21–32) days, respectively. The median dose intensity for patients randomised to Group A was 86 mg m^−2^ week^−1^ (range, 67–100) for CPT-11 and 598 mg m^−2^ week^−1^ (range, 296–667) for gemcitabine corresponding to 86 and 90% of the planned CPT-11 and gemcitabine doses, respectively. The median dose intensity of CPT-11 in patients randomised to Group B was 99 mg m^−2^ week^−1^ (range, 58–100), which corresponded to 99% of planned dose.

In total, 84 (29%) and 29 (12%) cycles were delayed in Groups A and B, respectively, (*P*=0.001). A total of 42 (14.4%) and 10 (4.1%) cycles in Groups A and B, respectively, (*P*< 0.001) were delayed by more than 7 days (median 11 days; range 8–41). Haematological toxicity was the main reason for treatment delay (Group A, *n*=35 cycles; Group B, *n*=4 cycles; *P*=0.006).

In all, 20 (7%) and 16 (6.6%) cycles of Groups A and B, respectively, required dose reductions (*P*=0.896), mainly because of haematological toxicity (Group A, *n*=9 cycles; Group B, *n*=1 cycle; *P*=0.018).

### Toxicity

The haematological and non-haematological toxicities are summarised in Table 3

**Table 3:** Haematological and nonhaematological toxicity of CPT-11+GEM and CPT-11 alone, as second-line treatment in NSCLC patients

	**CPT-11+GEM (Group A)**		**CPT-11 (Group B)**	
	**No. of pts**		**No. of pts**	
**Toxicity**	**All grades**	**Grade 3/4**	**All grades**	**Grade 3/4**
Haemoglobin	73 (26%)	6 (8%)	51 (68%)	—
Neutrophils	44 (58%)	21 (28%)	23 (32%)	13 (18%)
Platelets	39 (51%)	7 (9%)	12 (17%)	2 (3%)
Febrile neutropenia	3 (4%)	3 (4%)	8 (11%)	8 (11%)
Nausea/vomiting	26 (34%)	5 (7%)	22 (20%)	3 (4%)
Diarrhoea	31 (41%)	12 (16%)	37 (52%)	15 (23%)
Mucositis	3 (4%)	1 (1%)	3 (4%)	—
Asthenia	33 (59%)	6 (8%)	35 (49%)	9 (13%)
Allergic reactions	7 (9%)	—	—	—

. Grade 3 and 4 neutropenia occurred in 21 (28%) and 13 (18%) patients in Groups A and B, respectively (*P*=0.180). A total of 128 (44%) and 73 (30%) cycles in Groups A and B, respectively, required prophylactic rhG-CSF support (*P*=0.001). Febrile neutropenia occurred in three (4%) Group A and eight (11%) Group B patients (*P*=0.092); one patient in each arm died of sepsis despite treatment with broad-spectrum i.v. antibiotics and rhG-CSF support. Six (8%) patients randomised to Group A developed grade 3 and 4 anaemia compared with no such cases in Group B (*P*=0.029). A total of 28 (37%) and 19 (27%) Group A and B patients, respectively, who presented with haemoglobin values ⩽10.0 g dl^−1^, required at least one course of recombinant human erythropoietin administration. Seven (9%) patients of Group A and two (3%) patients of Group B developed grade 3 and 4 thrombocytopenia (*P*=0.106). No patients developed clinical bleeding episodes requiring platelet transfusions or hospitalisation.

Grade 3 and 4 nausea and vomiting were reported in five (7%) and two (3%) of the Group A and B patients, respectively. The incidence of diarrhoea (grade 2–4) was 25% in the combination and 40% in the monotherapy arm (*P*=0.169). Asthenia was equally distributed across the groups; grade 3 and 4 asthenia was reported in five (7.0%) and nine (13%) Group A and B patients, respectively (*P*=0.339). Severe asthenia was the reason for treatment discontinuation in one patient randomised to CPT-11. Other toxicities were mild (Table 3). There was no significant difference in grade 3 and 4 haematological or nonhaematological toxicities according to PS.

There were two Group A (one with grade 4 neutropenia+sepsis and one with grade 4 diarrhoea+hypovolaemic shock) and three Group B (one patient with grade 4 neutropenia+sepsis, one patient with sudden death occurring 9 days after the treatment administration and one patient with acute bowel obstruction and perforation) treatment-related deaths.

### Quality of life

In all, 108 (73.5%) (Group A, *n*=53 (69.7%); Group B, *n*=55 (77.5%)) patients completed and returned questionnaires for the evaluation of the impact of chemotherapy on the quality of life.

Comparison between the treatment regimens showed that patients treated with the combination regimen recorded better responses to all questions than patients treated with CPT-11 alone. For patients who had completed both evaluations for cycles 1 and 3 (*n*=80 patients), three of these differences indicated significant improvements for the questions about: ‘general mood today’ (*P*=0.014), ‘coughing’ (*P*=0.033) and ‘intensity of symptoms’ (*P*=0.034). Comparison of responses between cycles 1 and 6 for the 35 patients who had completed both evaluations revealed significant improvements in favour of the combination arm for questions about ‘coughing’ (*P*=0.003) and ‘general mood today’ (*P*=0.006). The global quality of life score showed a trend in favour of the combination arm in cycle 3 (*P*=0.097) but not in cycle 6 (*P*=0.313).

## DISCUSSION

The present multicentre, randomised, phase II study was designed to evaluate whether a combination chemotherapy regimen, using different anticancer drugs than those used in front-line treatment, is better than single agent as second-line treatment of patients with advanced NSCLC. Since the majority of patients had already received docetaxel–cisplatin first-line therapy, CPT-11 in combination with gemcitabine was investigated as second-line treatment (Kakolyris *et al*, 2002). The results clearly demonstrate that CPT-11 with gemcitabine resulted in a significantly higher objective response rate and improved quality of life compared with CPT-11, but failed to demonstrate any survival, duration of response and TTP advantage.

The observed antitumour activity of CPT-11 alone, which was in agreement with the results reported by Negoro *et al* (Ferrigno and Buccheri, 2000) was poor. Conversely, the response rate for the CPT-11/gemcitabine combination was satisfactory and significantly higher than the obtained with CPT-11 alone. In the Crino's study, gemcitabine was active as second-line treatment in patients with NSCLC (Crino *et al*, 1999). However, in this study almost half of the patients had stage IIIA or IIIB disease, and only 15% of them had received a taxane+platinum as first-line chemotherapy. Conversely, in our study, all patients had stage IV disease and had received docetaxel/platinum front-line chemotherapy. In addition, the median time interval from the last cycle of first-line chemotherapy was 3 months, suggesting that patients in the present study had a severe probability of having chemoresistant disease. Preclinical studies have demonstrated that there is a synergism between the two drugs as already demonstrated in preclinical studies (Pei *et al*, 1997; Tsuruo *et al*, 1998; Schwartz *et al*, 2001); however, this conclusion is difficult to drawn only on the basis of our clinical observations.

The median overall survival was not statistically different in patients treated with the combination regimen (9 months; 1-year survival: 24.5%) from those treated with CPT-11 alone (7 months; 1-year survival: 29%). Only PS was found to have a significant effect or survival (*P*=0.049), since the risk of death for patients with PS 2 was about twice that of patients with PS 0–1 (HR=1.853). It is interesting that the overall median survival observed with CPT-11 alone was numerically similar with that observed with single-agent docetaxel in the Shepherd's (Shepherd *et al*, 2000) and Fossella's (Fossella *et al*, 2000) studies. It would be of interest to compare CPT-11 as second-line chemotherapy and best supportive care in patients with advanced/metastatic NSCLC pretreated with docetaxel/platinum combination.

Until recently, the benefit of any second-line chemotherapy for the treatment of patients with inoperable stage IIIB and IV NSCLC was questionable. In 1997, the ASCO guidelines for second-line treatment of NSCLC stated that ‘second-line treatment may be appropriate for good PS patients for whom an investigational protocol is not available or desired, or for patients who respond to initial chemotherapy and then experience a long progression-free interval off treatment’ (Am Soc Clin Oncol, 1997) The results of the present study demonstrate no clear correlation between the response to first-line chemotherapy and the probability of response to second-line chemotherapy, since 12 responders to second-line chemotherapy experienced SD or PD to first-line chemotherapy; however, the low number of responding patients necessitates the cautious interpretation of the**se** results. The selection of patients with advanced NSCLC who might derive the greatest benefit from second-line chemotherapy remains important. In a prospective randomised trial of second-line docetaxel *vs* best supportive care, it could be demonstrated that patients with weight loss greater than 10%, LDH, multiorgan or liver involvement did not benefit from second-line chemotherapy; conversely, refractoriness to front-line platinum-based chemotherapy is not an independent prognostic factor (Shepherd *et al*, 2001).

The optimal chemotherapy regimen for the second-line treatment of advanced NSCLC remains elusive. Single-agent paclitaxel (Socinski *et al*, 1999), docetaxel (Fossella *et al*, 2000; Shepherd *et al*, 2000) and gemcitabine (Baas *et al*, 1999; Rossi *et al*, 1999) have been investigated in the second-line setting. In addition, several phase II studies have evaluated different drug combinations in the second-line setting (Androulakis *et al*, 1999; Herbst *et al*, 1999; Herrero *et al*, 2000; Kosmas *et al*, 2001; Leu *et al*, 2001; Laack *et al*, 2002) The data from these studies strongly suggest that, although response rates may be higher when a combination regimen is used second line, the median overall survival ranges from 6 to 8 months, which is not significantly different from that observed with single agents.

In the present study, both single-agent CPT-11 and the CPT-11 in combination with gemcitabine were relatively well tolerated. It is interesting that the toxicity profile of each treatment was not very different in patients with PS 0–1 and 2. However, a significantly higher number of chemotherapy cycles in the combination than in the single-agent arm had to be delayed for more than 7 days mainly due to haematological toxicity. The higher incidence, although nonstatistically significant, of grade 3–4 neutropenia in the combination arm as well as the increased incidence of febrile neutropenia in the monotherapy arm should be attributed to the more common use of rhG-CSF in the combination regimen. There was one treatment-related death due to sepsis in each arm. The other toxicities were mild and there was no significant difference in their incidence between the two treatment arms.

In conclusion, our results failed to demonstrate a survival advantage of CPT-11+gemcitabine over CPT-11, although the combination regimen was proved superior in terms of response rate and quality of life. Recently, Fossella *et al* (2003) reported that the docetaxel+CDDP regimen was superior in terms of overall survival than the vinorelbine+CDDP combination. Therefore, an increasing number of patients will be treated with front-line docetaxel and cisplatin; for the patients who relapse after an initial response or progress while receiving front-line docetaxel+CDDP, the CPT-11+gemcitabine combination is a reasonable second-line therapeutic option. Whether the combination of second-line CPT-11/gemcitabine and sequential administration of EGFR tyrosine kinase inhibitors could improve overall survival of patients with advanced/metastatic NSCLC remains an attractive question.

## Appendix

The participating centres and investigators is provided in Table A1

**Table A1:** Participating centres and investigators

**Centre**	**Investigators**
Dpt of Medical Oncology, University General Hospital of Heraklion, Crete	V Georgoulias, Ch Kouroussis, D Mavroudis, N Androulakis
1st Dpt of Pulmonary Diseases, Hospital of Chest Diseases of Athens	A Agelidou, E Papadakis
2nd Dpt of Pulmonary Disease, ‘Sismanoglion’ General Hospital of Athens	F Apostolopoulou, X Tsiafaki, M Agelidou
2nd Dpt of Medical Oncology, ‘Theagenion’ Anticancer Hospital, Thessaloniki	I Boukovinas, K Dimitriadis, I Stergiou, P Macrantonakis
1st Dpt of Pulmonary Diseases, ‘Sigmanoglion’ General Hospital of Athens	Ph Palamidas, P Ziotopoulos
1st Dpt of Medical Oncology, ‘Agios Savvas’ Anticancer Hospital of Athens	A Ardavanis, E Stavrinidis, G Rigatos, A Alexopoulos
8th Dpt of Pulmonary Diseases, Hospital of Chest Diseases of Athens	O Anagnostopoulou, G Pavlakou, A Rapti
Medical Oncology Unit, ‘Laiko’ Hospital of Athens	A Polyzos
Medical Oncology Unit, Dpt of Internal Medicine, Hospital of Pulmonary Diseases of Athens	K Syrigos
Dpt of Pulmonary Diseases, ‘Papanikolaou’ General Hospital of Thessaloniki	N Samaras
Dpt of Pulmonary Diseases, University General Hospital of Heraklion	N Tzanakis
2nd Dpt of Medical Oncology, ‘Agii Anargyri’ Anticancer Hospital of Athens	Th Giannakakis, S Papadouris
3rd Dpt of Pulmonary Diseases, Hospital of Chest Diseases of Athens	F Christou
7th Dpt of Pulmonary Diseases, Hospital of Chest Diseases of Athens	M Toubis
Medical Oncology Unit, 401 Military Hospital of Athens	Ch Christophylakis
Dpt of Medical Oncology, General Hospital of Larissa	A Athanasiadis
Dpt of Pulmonary Diseases, University of Athens, ‘Sotiria’ Hospital, Athens	M Veslemes
Dpt of Internal Medicine, ‘Patision’ General Hospital of Athens	E Tselepatiotis
